# Electronic Health Interventions in the Case of Multiple Sclerosis: From Theory to Practice

**DOI:** 10.3390/brainsci11020180

**Published:** 2021-02-02

**Authors:** Maria Scholz, Rocco Haase, Dirk Schriefer, Isabel Voigt, Tjalf Ziemssen

**Affiliations:** Center of Clinical Neuroscience, Department of Neurology, University Hospital Carl-Gustav Carus, Dresden University of Technology, 01307 Dresden, Germany; maria.scholz@uniklinikum-dresden.de (M.S.); Rocco.Haase@uniklinikum-dresden.de (R.H.); Dirk.Schriefer@uniklinikum-dresden.de (D.S.); Isabel.Voigt@uniklinikum-dresden.de (I.V.)

**Keywords:** multiple sclerosis, digital health, eHealth, intervention, patient management

## Abstract

(1) Background: eHealth interventions play a growing role in shaping the future healthcare system. The integration of eHealth interventions can enhance the efficiency and quality of patient management and optimize the course of treatment for chronically ill patients. In this integrative review, we discuss different types of interventions, standards and advantages of quality eHealth approaches especially for people with multiple sclerosis (pwMS). (2) Methods: The electronic databases PubMed, Cochrane and Web of Science were searched to identify potential articles for eHealth interventions in pwMS; based on 62 articles, we consider different ways of implementing health information technology with various designs. (3) Results: There already exist some eHealth interventions for single users with a single-use case, interventions with a social setting, as well as eHealth interventions that integrate various single and social interventions and even those that may be used additionally for complex use cases. A key determinant of consumer acceptance is a high-quality user-centric design for healthcare practitioners and pwMS. In pwMS, the different neurological disabilities should be considered, and particular attention must be paid to the course of the treatment and the safety processes of each treatment option. (4) Conclusion: Depending on the field of application and the respective users, interventions are designed for single, social, integrated or complex use. In order to be accepted by their target group, interventions must be beneficial and easy to use.

## 1. Introduction

**Figure 1:**
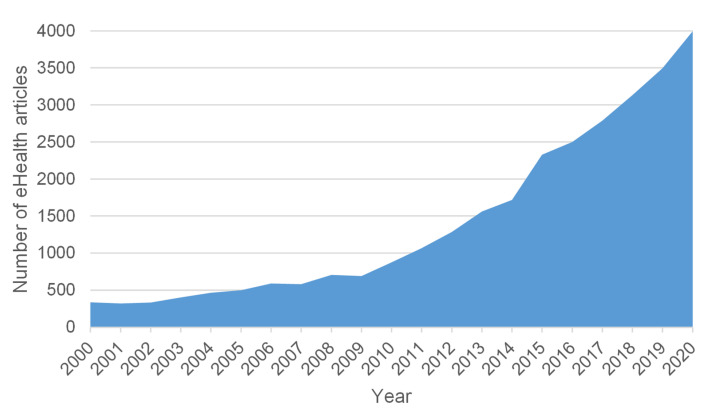
Publication trend: number of publications on the search query of eHealth interventions over the last two decades at PubMed.

In a society of growing digital proficiency, 80 percent of all Internet users go online to seek health information [1]. The use of health information technology (HIT) in healthcare has become increasingly prominent since the late 1980s [2]. Early HIT mainly referred to the digitization of traditional processes in the public health sector. With the development of new technologies, the term has become more general [3]. Focusing on eHealth-assisted patient management, we have also witnessed a steady increase of research interest in the last two decades (Figure 1) [4].

**Figure 2:**
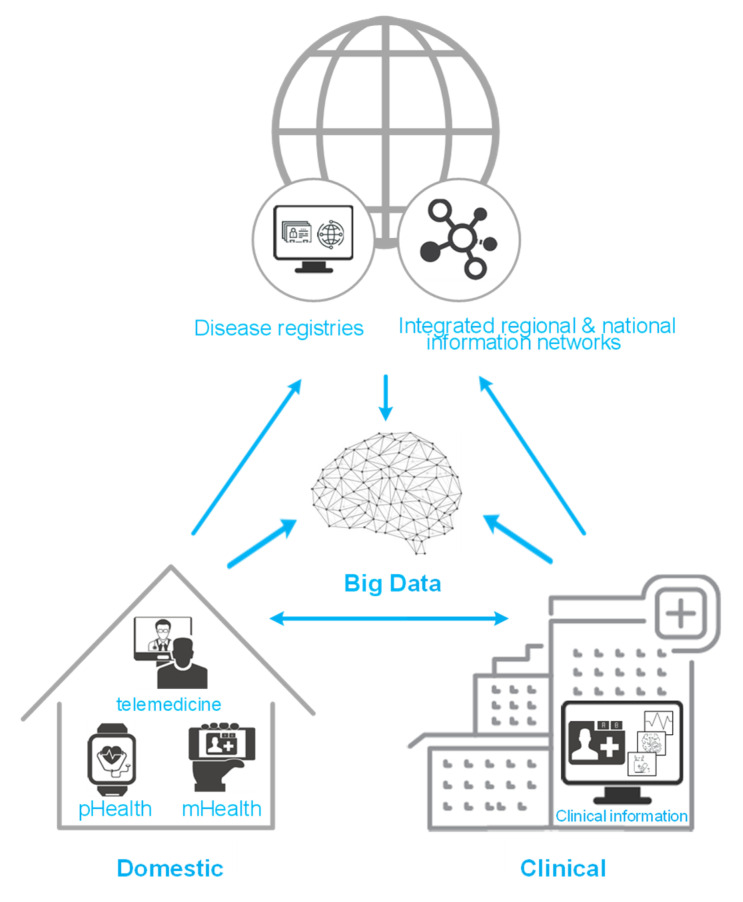
Different eHealth technologies used in domestic settings such as personalized health (pHealth) and mobile health (mHealth), clinical settings or both for collecting or presenting patient data and their interaction possibilities with each other. Clinical information represents all data collected in the clinical environment together yielding the electronic health record.

Several approaches exist to defining constructs such as eHealth, telehealth and other HIT terms [5], but we want to provide a common ground for our review: eHealth is defined as “an emerging field in the intersection of medical informatics, public health and business” using information and communication technologies [3]. Such technologies are shown in Figure 2 and may contain personalized health (pHealth), telemedicine and telecare, mobile health (mHealth), clinical information systems (e.g., electronic health record), disease registries and other non-clinical systems, integrated regional and national information networks and Big Data approaches [2,6,7].

Good eHealth interventions are easy to use and should enhance efficiency and quality, translate evidence-based knowledge into practice, enable patient empowerment by giving them more control over their health, education and information exchange as well as facilitate specific interventions [8,9,10]. Especially for people with chronic diseases, adequate treatment and monitoring are difficult to supply [11]. eHealth interventions are an effective way to identify the health needs of people with complex chronic diseases and may meet their long-term care needs because many areas can be addressed and acceptance as well as satisfaction with such interventions is supposed to be high [12].

An important chronic disease is multiple sclerosis (MS), one of the world’s most common neurological disorders of young adults that results in central demyelination and neurodegeneration causing multifocal neurological problems [13,14,15,16]. Usually, people with MS (pwMS) show their first symptoms at the age of 20 to 40 years; consequently, they live with this chronic disease for the following decades, which is why these patients may be important early adopters of emerging eHealth trends [17]. Additionally, their physical and cognitive impairments complicate traditional face-to-face interventions for pwMS. Such disabilities and the willingness to use digital media for communication with healthcare providers make MS an excellent model for innovative improvements in care delivery, including eHealth interventions [18,19,20]. In addition to individual disabilities, there are other circumstances that make a face-to-face visit even more challenging. On the one hand, these include geographical barriers. Long distances to specialists, especially in rural areas, mean an enormous effort for patients to obtain the required care. On the other hand, there are special situations such as the recent coronavirus disease (COVID-19) pandemic making smooth patient care problematic. Reducing person-to-person contact in order to stop a rapid spread of the disease is a preventive measure against proliferation [21]. This commandment and the fear of possible infection lead patients to cancel their medical appointments. To enable continuous patient care without a face-to-face visit and to overcome geographical barriers [22], eHealth interventions can serve as a helpful tool. By extending the collection of health data electronically beyond the consultation itself, a continuous recording of all facets of this complex disease may enable a safe and efficient management of the individual disease course. Therefore, HIT serves as a support for medical and health policy practice [11] that reduces costs. Since the quality of care achieved by eHealth interventions may deviate from traditional face-to-face interactions, cost reductions and treatment outcomes need to be balanced. To optimize the specific treatments of pwMS, eHealth interventions can support physicians in long-term documentation and management of treatment steps in any disease-modifying therapy (DMT) [23]. 

The aim of this integrative review is to offer an overview of eHealth interventions for pwMS grouped into single, social, integrated or complex eHealth interventions. We also provide not only a theoretical description of the benefits of complex interventions, but also a practical demonstration. Key factors for a successful development of good patient management are discussed.

## 2. Materials and Methods

We searched the electronic databases PubMed, Cochrane and Web of Science from 2000 to December 2020 to identify potential articles for eHealth intervention in pwMS. The keywords used in this article were “(eHealth OR telemedicine OR telehealth OR “digital health” OR “mobile Health” OR “personalized Health” OR “electronic health”) AND “multiple sclerosis” NOT (Parkinson OR “major depressive disorder” OR epilepsy OR diabetes)”. In total, there were 451 articles obtained from the three databases using the keyword searches. After looking for false entries that did not focus on MS and articles that were not written in English or German, 283 articles were determined to be irrelevant to this study and 106 were recognized as duplicates and were therefore removed. The remaining 62 articles were reviewed and discussed. As a taxonomy for eHealth interventions, we consider different ways to implement HIT, for instance, by phone, telehealth, web-based, via remote sensoring or virtual technologies [7,24,25], which are designed as an intervention with a single-use case, interventions with a social setting, as well as an eHealth intervention that integrate various single and social interventions and even those that may be used additionally for complex use cases. There are numerous heterogeneous options for classifying eHealth interventions [26,27]. In this integrative review, we applied a classification based on a taxonomy that was developed for the Office for Life Sciences of the UK [28,29]. For some interventions, we provide information for cooperation and funding in brackets. Additionally, we present some success factors of eHealth interventions for pwMS found in the selected literature.

## 3. Results

### 3.1. eHealth Interventions for a Single-Use Case

Health technologies for a single-use case focus on a single purpose for an individual user. Typically, single-use interventions are consumer-initiated and record vital measurements, training values, health behavior, medication and food intake [8,11]. In this way, pwMS use applications that focus on their disease. Patients can record their medication intake, subsequent consultations and disease-related vital signs to get an overview of their disease progression. It is also feasible to inform pwMS about the latest results and guidelines for different MS treatments and medications via single-use technologies or to remind them of appointments or medication intake [18,30,31]. 

The Multiple Sclerosis Centers of Excellence website (Veterans Health Administration; 2003) is such a single-use application for an eHealth intervention. It prepares caregivers for the special needs of MS and empowers patients to care for themselves by asking questions and providing guidelines on the website [32]. Self-managing MS is feasible with MS Energize (AUT Ventures, New Zealand; New Zealand; Kiwinet, New Zealand; MEA Mobile, Stuttgart, Germany; 2019) [33], MSCopilot (AD SCIENTIAM, Paris, France; 2019) [34], MS COMPASS++ (PEARS HEALTH CYBER, Czech Republic; 2015) [35], via digital diary [36], MS Invigor8 [37], Managing Fatigue [38], MS Sherpa (MS sherpa BV, Nijmegen, Netherlands; 2019) [39], MyMS&Me (Irody, Inc., Boston, MA, USA; 2020) [40] and other mobile applications [41]. Especially wireless and wearable devices are useful interventions to enhance rehabilitation in pwMS [26,42]. Functions of the autonomic nervous system, upper and lower limb functions, movement, cognition and other body functions can be permanently recorded with accelerometers, gyroscopes and glove-type monitors and thus provide a more precise overview of the disease [26]. Special offers such as Home-Based Tablet App for Dexterity Training (Swiss Multiple Sclerosis Society, Bayer AG; 2020) [43], the personalized mobile application WalkWithMe [44], the App for Dual-Task Assessment and Training Regarding Cognitive-Motor Interference (Novartis Pharma AG;; Swedisch PROMOBILIA foundation; Flemish MS Liga; 2016) [45] and additional eHealth interventions [46,47,48,49,50,51,52] offer exercises to reduce various disabilities.

### 3.2. Social eHealth Interventions

The availability of supporters can help chronically ill patients to face their daily challenges and improve their self-management in chronic diseases. Social eHealth interventions can provide social support from other users such as experts, physicians or other patients [8]. Social eHealth interventions enable these physically and cognitively impaired people to participate digitally in communities and stay “connected” with friends and family [53]. With the application of gamification, patients are encouraged and motivated to make greater use of eHealth interventions and achieve goals more easily [8]. Gamification is the application of game design elements such as rewards, challenges and competition, teamwork, point scoring and rankings [54].

Social media such as the BartsMS Blog, the SMsocialnetwork [26], PatientsLikeMe [55], the Overcoming Multiple Sclerosis [56] website and telemedicine support such as My Support Plus [57], ECHO [58], CareCall [59], Tele-MIT [60], Multiple Sclerosis at Home Access [61] and *Télé-SEP* [62] try to prevent misinformation, disseminate valid information and improve quality of care. Patients can talk about their illness or ask specialists for advice before their visit. The T-EDSS is a telephone-based Expanded Disability Status Scale) (EDSS) that simplifies communication for physicians and patients by eliminating the need to commute to health centers [63]. YouTube videos created by pwMS are used for communication (e.g., dealing with blindness or chronic illness in daily life) and education between patients [64]. However, patients should be careful as long as all information is not regularly checked for accuracy and correctness. In addition to online offers, gamification is a playful way to train physical and cognitive areas, even if it does not replace telerehabilitation [26]. To improve sensory strategies, pwMS can use gamification intervention such as Nintendo^®^ Wii^®^ Balance Board^®^ (Nintendo, Kyoto, Japan), Xbox 360^®^ (Microsoft, Redmond, WA, USA) and Kinect console (Microsoft, Redmond, WA, USA) [65,66], More Stamina [67] or BrainHQ^®^ tool (PositScience, San Francisco, CA, USA) [68]. Visual feedback exercises can be used to train balance [69]. To improve functional outcomes, home-based physical telerehabilitation [15] or web-based telephone consultations like FACETS [70] have become a useful complement to standard interventions.

### 3.3. Integrated eHealth Interventions

Integrated eHealth interventions link patients with the healthcare system. Apps and websites are used to provide information exchange between healthcare providers, deliver video and image instructions to patients and encourage them speaking about their experience with the disease and the way they deal with it. This is an optimal way to prepare caregivers for the special requirements of MS [32]. It is also applied to remind participants to take medication and monitor their compliance, eating habits and emotional well-being. Furthermore, integrated eHealth is used to send educational and motivational messages or to provide feedback to patients and to support them in self-managing their chronic condition [8,11,32,53].

The web-based Mellen Center Care On-Line (MCCO) (Cleveland Clinic., Cleveland, OH, USA; 1998) [71] patient portal provides improved patient–physician communication, information about the disease through appropriate links and control over disease progression as well as future clinical visits [18]. Other integrated eHealth interventions that simplify and control clinical procedures or reduces costs and geographical barriers are “GP at Hand” (Babylon GP at Hand, London, UK; 2021), MS Mosaic (Duke Health and Duke University; 2004), Floodlight (Genentech, Inc., Basel, Switzerland; 2021), ElevateMS (Sage Bionetworks, Novartis Pharmaceuticals Corporation, Seattle, WA, USA; 2017), MSmonitor (TEVA Netherlands; 2014), PatientConcept (NeuroSys GmbH, Ulm, Germany; 2015), PatientSite (Beth Israel Deaconess Medical Center, Boston, MA, USA; 2000), [9,17,26,72,73] the Open MS BioScreen [74], MS PATHS (Biogen, Cambridge, UK; 2020) [75], MSProDiscuss (Adelphi Communications Ltd.; Novartis, London, UK; 2020) [76] and Multiple Sclerosis Documentation System (MSDS)^3D^ (MedicalSyn GmbH, Stuttgart, Germany; 2010) [77]. MSDS^3D^ can not only be used for the collection and interpretation of patient data, but also to monitor drug safety as well as for conveying information to the patient and to get a clinical opinion from an expert neurologist or radiologist via the expert advice tool of MSDS^3D^ [78,79].

### 3.4. Complex eHealth Interventions

Complex eHealth interventions have multiple components for interaction. They focus on the optimal management of a particular disease. Data, collected electronically by patient, physician or nurse, are analyzed by the system and used for the prognosis of the chronic disease. This enables the early recognition of critical events and correlations in social processes. Furthermore, the interpretation of complex data by the system enables a quick prediction of answers to various questions [80]. These systems increase safety and control the efficacy of MS therapies [26,27]. The next step for MSDS^3D^ will be the inclusion of specific management pathways. The implementation of such clinical pathways for disease monitoring or the treatment of symptomatic disabilities will enable data-driven standardized care and make it measurable and verifiable [81]. The quality of care can be assessed implementing guidelines [23] and pharmacoeconomic outcomes can be analyzed as well [82]. 

The Home Automated Tele management (HAT) system is such an intervention that analyzes patient self-testing results and reviews computer-generated alerts. It implements computerized decision support based on individualized alert setup and real-time monitoring of patient self-testing data. A personalized training plan with written descriptions and a video of the therapist performing the exercise is uploaded to a HAT home unit for each patient [83]. Another intervention is the MS SCDS toolkit that facilitates quality initiatives and ensures that care conforms to best practices. This toolkit supports initial and follow-up visits [84]. 

**Figure 3:**
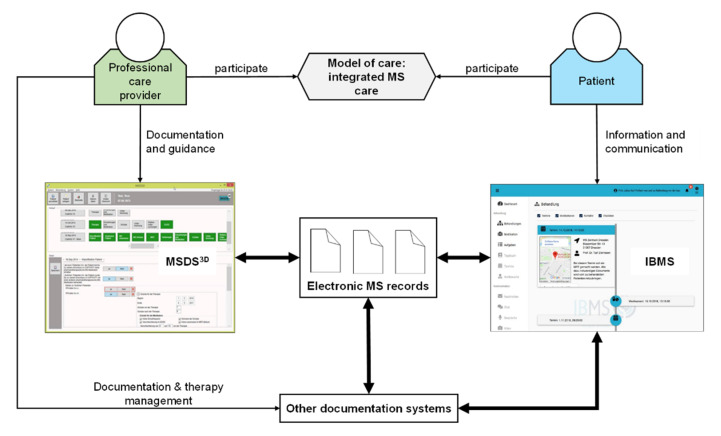
eHealth application of informal care, example of a basic concept, adapted from [85]. Multiple sclerosis (MS); Multiple Sclerosis Documentation System^3D^ (MSDS^3D^); Integrated Care Portal Multiple Sclerosis (IBMS).

The Integrated Care Portal Multiple Sclerosis (IBMS) is an eHealth portal solution that is adapted to the clinical patterns of MS and associated patient needs to improve the overall diagnostic and therapeutic quality of care [85]. Therefore, IBMS is connected to the existing MSDS^3D^ and enables fast and easy networking of all participating healthcare providers. Necessary medical diagnostic services such as magnetic resonance imaging (MRI) or laboratory examinations are accessible more quickly from any location. Therapeutic decisions are supported by experts and implemented efficiently and in accordance with guidelines. Information on examination results, previous illnesses or medication can be read into an electronic patient record that is accessed by different physicians any time so that they can quickly and purposefully consider interdisciplinary patient information for treatment (see Figure 3 for the basic concept of IBMS). Patients with difficult disease progression and complex care requirements can be assigned to the specialized setting of the University Hospital Dresden in order to guarantee the best possible care being tailored to their needs. Patients with less complex care needs can be treated locally by general practitioners or neurologists in private practices for routine presentations or information meetings. This efficient demand-oriented use of health services is intended to reduce the physician workload and treatment costs. 

In addition to the integration of professional healthcare providers in the treatment of MS, IBMS has set itself the goal of improving the integration of patients and their families. Patients and their family (if requested by the patients) should also be able to view diagnostic results and other medically relevant data on their treatment in a comprehensible form. Moreover, pwMS and their family can receive recommendations for treatment based on the latest guidelines. In this way, the willingness of the family to participate in the coordination of patient care can be improved and the patients have a stronger involvement in their treatment management. Obstacles in treatment management are distance to healthcare, complex clinical patterns or job constraints. The use of modern information and communication technologies must make treatment as independent of time and place as possible in order to overcome such obstacles. This reduces not only job-related restrictions and the associated costs but also strengthens the mental resources of family members.

### 3.5. Success Factors of eHealth Interventions for pwMS

After looking through the existing landscape of eHealth interventions for pwMS, we identified general factors of success and failure that can influence the use of eHealth interventions. A key determinant of consumer acceptance and engagement with these programs is a high-quality user-centric design [86]. This includes the accommodation for varying physical and cognitive impairments and providing high-quality information, choice and control as part of overcoming practical challenges [87]. 

Visual deficits require a large font with large line spacing, low contrast to black letters on a white background, no color and no blinking effects for the interventions. Because of possible motor impairment, there must be an alternative for control beyond mouse or keyboard. One possibility would be the linguistic control or using only a few keyboard buttons. Cognitive limitations can be bypassed by creating an intuitive user interface [18]. To improve walking impairments and avoid comorbidities such as cardiovascular disease, eHealth interventions should enhance the physical activity [88]. 

In order to provide pervasive and patient-centered care, the design of interventions should be appropriate and tailored both for healthcare practitioner and for patients [10,11]. Individual counselling, group contacts and self-management also increase the use of eHealth applications [7,8,10]. 

Social support can increase empowerment and self-management skills and motivate chronically ill people like pwMS to search for health information online [7,8,89,90]. Furthermore, gaming-based systems such as the Nintendo^®^ Wii^®^ Fit console or Kinect motion sensor motivate patients to use eHealth applications [1,9]. The adaptation of digital self-monitoring tools to a patient’s personal situation, guidance to increase the value of the data and integration of digital self-monitoring into treatment plans are features that can increase user acceptance [91]. The design of eHealth interventions must be tailored to different users and consider a wide range of aspects. Therefore, it is necessary to design flexible interfaces. Especially chronically ill patients like pwMS need an adaptive design for various symptoms. EHealth interventions are not only used for rehabilitation but also for preventing risk behaviors [1,4,8,9,53]. To meet all needs and demands of pwMS, healthcare professionals, researchers and industry partners must work together to develop effective eHealth solutions [92].

In addition to the design requirements and the consideration of different user perspectives, application support plays an important role in the uncomplicated implementation of interventions. A lack of knowledge about new technologies and programs by patients and even professional nurses makes it harder to use the applications without errors. Therefore, training on such technologies and programs for caregivers and patients is required in order to provide easy access and act in accordance with predefined protocols. Continuous data recording and user acceptance of an intervention can only be achieved if device and system errors as well as technical difficulties in uploading self-monitored data are avoided or promptly remedied. In order to ensure a legally secure consultation between patient and healthcare professional as well as sound patient management, an appropriate standard of care must be achieved [93,94,95]. This means, on the one hand, protecting electronic data from misuse and manipulation and, on the other hand, interconnecting different systems and making data transparent [24,96]. A major challenge remains to ensure interoperability between different types of systems and data sources [2]. The basis for successful data exchange between participating individuals is the assurance of standards for data protection and data security. Informations critical to the individual should only be collected, stored or disseminated following their guidelines. In this context, it must be transparently defined what kind of data are collected and how they are stored, who ultimately owns them, how the patient can access them, and who gets (partial) access to them [97]. Especially in industry-funded projects, different interests clash when it comes to who gets access to study data, as well as when and how. Significant progress has been made in this area in recent years, not the least due to the European General Data Protection Regulation. However, the more complex the scope and the larger the group of the addressed audience, the higher the requirements are for establishing a (multinational) eHealth project as well as those for monitoring compliance with existing regulations. In principle, integrated and complex eHealth interventions offer a more promising approach here as opposed to a plethora of individual apps, as a smaller number of bundled data protection processes enables a more careful examination of the respective conditions through the patient. Likewise, longer support times and more easily reachable responsible entities can be expected for data protection inquiries in larger projects [98].

## 4. Discussion

EHealth interventions are helpful tools to close the supply shortfall in the healthcare system and to improve the care of chronically ill patients because they can present the course of illness more comprehensively and more accurately than face-to-face visits. They are designed for various use cases and different users. On the one hand, individual health parameters of patients can be entered and interactions with physicians or other patients can take place. On the other hand, health systems are interconnected to exchange data, give feedback and receive optimal disease management. For the implementation of eHealth interventions, a number of requirements for various deficits in relation to different diseases must be considered. Well-designed interventions can provide relief to the patient and all other persons involved in the recovery process if the digital divide in chronic care can be minimized [24]. 

PwMS may be an ideal, trend-adopting group of eHealth users. There are already several eHealth interventions on the market for MS, specifically targeted to the impairments of the disease. One example is the MSDS^3D^. A special feature of MSDS^3D^ is the focus on the management of individual DMTs, which ensures a comprehensive and safe treatment of the patient. The system also includes a module focusing on treatment satisfaction. Treatment satisfaction is an important factor for patient compliance and an indication of comorbidity; therefore, it should be added to any eHealth intervention directly [36]. Being connected to MSDS^3D^ via the IBMS portal, both physicians and patients are able to follow the course of disease exactly. In addition, patients and their family can exchange information with healtcare professionals via the platform and obtain the latest information. 

Our research is not without limitations. In the present review, only those papers published in English or German in PubMed, Cochrane or Web of Science were included. This may have resulted in the omission of eHealth interventions from other databases, in other languages, without a product name or not (freely accessible) published commercial interventions of private companies. Our definition of eHealth has also led us not to include domains such as robotics. In this paper, we only presented various interventions and mentioned their positive aspects. However, we did focus only on the effect of eHealth interventions on treatment or patient management. It is to be determined whether eHealth interventions help to provide a better picture of disease status than standard interventions and whether these technologies are associated with improvements in long-term patient outcomes. It is important to know how effective the individual interventions are and to identify the most useful and cost-effective technology. This could increase acceptance of the use of eHealth interventions. However, the comparison of the interventions is difficult due to the differently used methods. In addition, long-term studies are necessary to determine the long-term effect of an intervention, but for cost reasons, this is rarely done. It would also be informative to know how interventions can assist the paradigm shift of healthcare from disease-focused to patient-centered and facilitate conducting pragmatic clinical trials.

Future research should focus on patients’ self-monitoring to empower them in viewing and understanding their disease progression independently of the physician and in making self-determined decisions regarding treatment. For this, interventions should not only be able to display data and results in an easy-to-understand manner but should also enable specific treatment options for each outcome (e.g., specific exercises for foot drop; different medication options). An option to make appointments with specialists would complement the intervention. This all leads us to a system that combines all health-related aspects in a patient- centered eHealth approach.
